# Nutritional Support for Exercise-Induced Injuries

**DOI:** 10.1007/s40279-015-0398-4

**Published:** 2015-11-09

**Authors:** Kevin D. Tipton

**Affiliations:** Health and Exercise Sciences Research Group, University of Stirling, Cottrell Building, Stirling, FK9 4LA Scotland, UK

## Abstract

Nutrition is one method to counter the negative impact of an exercise-induced injury. Deficiencies of energy, protein and other nutrients should be avoided. Claims for the effectiveness of many other nutrients following injuries are rampant, but the evidence is equivocal. The results of an exercise-induced injury may vary widely depending on the nature of the injury and severity. Injuries typically result in cessation, or at least a reduction, in participation in sport and decreased physical activity. Limb immobility may be necessary with some injuries, contributing to reduced activity and training. Following an injury, an inflammatory response is initiated and while excess inflammation may be harmful, given the importance of the inflammatory process for wound healing, attempting to drastically reduce inflammation may not be ideal for optimal recovery. Injuries severe enough for immobilization of a limb result in loss of muscle mass and reduced muscle strength and function. Loss of muscle results from reductions in basal muscle protein synthesis and the resistance of muscle to anabolic stimulation. Energy balance is critical. Higher protein intakes (2–2.5 g/kg/day) seem to be warranted during immobilization. At the very least, care should be taken not to reduce the absolute amount of protein intake when energy intake is reduced. There is promising, albeit preliminary, evidence for the use of omega-3 fatty acids and creatine to counter muscle loss and enhance hypertrophy, respectively. The overriding nutritional recommendation for injured exercisers should be to consume a well-balanced diet based on whole, minimally processed foods or ingredients made from whole foods. The diet composition should be carefully assessed and changes considered as the injury heals and activity patterns change.

## Introduction

Injuries are an inescapable aspect of exercising and participation in sport. The particular results of an exercise-induced injury may vary widely depending on the nature and severity of the injury. Injuries typically result in cessation, or at least a reduction, in participation in sport and decreased physical activity. More severe injuries may result in immobilization of a limb. Recent evidence suggests that half of the total number of injuries can be considered severe, leading to an average of >3 weeks without training or competing [1]. Thus, interventions that can increase the rate of healing and decrease the time to return to play are important. Among other options used by trainers, physicians and athletes, nutritional support may help enhance recovery. A great deal of material has been written on the topic of nutrition for exercise-induced injuries [2–4], but very little stems from studies directly examining these issues. The aim of this review is to examine and update the evidence for nutritional strategies to support the enhancement of recovery and return to training and competition. Given the relative dearth of direct information on nutrition for exercise-induced injuries, an attempt also will be made to glean what insight is possible from other models, including trauma, wound healing, immobilization and bed rest studies.

Most injuries severe enough to result in immobilization and/or reduced physical activity may be considered to have two main stages. Both stages may be influenced by nutrition. The first stage is the healing and recovery phase. Immediately after an injury, wound healing begins. It is a complex process involving three, overlapping phases: inflammation, proliferation and remodelling. Bone repair is similar to, but slightly different from, soft tissue repair [2]. This healing stage may involve reduced activity or even complete immobility of a limb lasting from only a few days up to several months depending on the nature and severity of the injury [5]. The second stage to consider follows the return to activity. Rehabilitation and increased activity, overall and for an immobilized limb, are typical of this stage. This second stage is much more clearly demarcated for injuries involving immobilization, but the transition between stages is less clear for other injuries. Typically, complete recovery and return to full function and training takes longer than the immobilization period [6]. Full recovery from some injuries may take even up to several years [7–9]. Thus, nutritional support may be crucial to lessen the length of time and reduce the negative aspects of reduced activity and immobilization, as well as to support the return to activity and training. Given that nutritional recommendations for increasing muscle size and strength during rehabilitation would be similar to other muscle growth situations [10–14], the focus of this review primarily will be on the first stage of injury, i.e. wound healing and reduced activity or immobilization. The bulk of this review will address injuries requiring immobilization and reduced physical activity, but there will also be discussion of nutrition for other injuries.

## Inflammation

During the first stage following an injury, an inflammatory response is initiated. The inflammatory response initiates activation of many processes that are crucial for optimal healing [15, 16]. This inflammation may last for a few hours up to several days depending on the type and severity of the injury [17]. An oft-cited aim of post-injury nutrition is to reduce, or even abolish, the inflammatory response. Excess inflammation is, of course, counterproductive for healing. However, given the importance of the inflammatory process for wound healing [17], a drastic reduction of inflammation may not be ideal for optimal recovery. Most exercise-induced injuries, particularly in otherwise healthy exercisers and athletes, would not be severe enough for uncontrolled inflammation to be an issue [17]. Thus, nutritional interventions intended to reduce inflammation [18–20] may be contra-indicated. Therefore, careful consideration of the appropriate approach to managing inflammation is important for optimal recovery from injury.

## Injuries Involving Immobilization and/or Reduced Activity

Injuries severe enough to result in immobilization of a limb and/or bed rest leading to drastically reduced levels of physical activity have obvious negative ramifications. Disuse of a limb results in loss of muscle mass and reduced muscle strength and function [6, 21–24]. Moreover, immobilization is detrimental for tendon structure and function [25]. Substantial muscle loss has been reported in as little as 5 days of disuse [24]. Earlier we reported that metabolic measurements in muscle suggest that muscle tissue is lost with only 36 h of inactivity [26]. Moreover, Reich et al. [27] reported altered gene expression with 48 h of muscle disuse. Thus, even injuries that result in only short-term muscle disuse may have negative metabolic consequences. Clearly, nutritional measures that may influence the response of muscle and tendon to injury-induced immobilization and inactivity can help an exerciser return to full activity and training more quickly.

The metabolic mechanism for changes in muscle mass is net muscle protein balance (NBAL), i.e. the balance between the rate of muscle protein synthesis (MPS) and breakdown (MPB). In particular, muscle size and strength are lost when there is negative balance between myofibrillar MPS and MPB. Muscle is lost over any given period of time when periods of negative NBAL are greater than periods of positive NBAL. During muscle disuse, the basal, i.e. resting and fasted, rate of MPS is decreased [21, 23, 28]. The influence of MPB on NBAL during muscle disuse is less clear. The measurement of MPB in humans is difficult, and indirect measures are often necessary to attempt to assess changes in MPB that may contribute to muscle loss. After 14 days of strict bed rest, dynamic measurement of MPB using stable isotopic tracers showed that MPB was decreased, albeit to a lesser extent than MPS [21]. Thus, after 14 days the decrease in MPS was greater than that of MPB leading to negative NBAL and muscle loss during disuse. There is now preliminary and indirect evidence that MPB may be increased during the first few days of immobility [24, 29, 30]. These data suggest that it is possible that a transient increase in MPB contributes to muscle loss early after a limb is immobilized, but increases in these indirect, static markers of MPB do not persist for longer periods, e.g. 14 days [31, 32]. Moreover, there is convincing evidence that static, indirect markers of MPB do not represent the dynamic muscle metabolism [33]. Thus, solid conclusions regarding the importance of MPB for muscle loss during limb immobility are lacking. Nevertheless, it seems clear that decreased MPS is the major metabolic mechanism behind negative NBAL and muscle disuse atrophy [28].

Another metabolic contributor to muscle loss with immobility is the resistance of MPS to anabolic stimulation. Both bed rest [34] and limb immobilization [23, 35] models demonstrate that muscle exhibits ‘anabolic resistance’ with disuse. The response of MPS to hyperaminoacidaemia from amino acid infusion [23], essential amino acid (EAA) ingestion [34], and protein ingestion [35] is reduced following a period of disuse. Moreover, complete muscle disuse is not necessary to stimulate some level of anabolic resistance. Simply reducing muscle activity for 14 days is enough to reduce the response of MPS to ingested protein [36]. The mechanisms for this lack of response to hyperaminoacidaemia are undoubtedly multifactorial and have yet to be definitively determined. Possible mediators of anabolic resistance with muscle disuse include impaired protein digestion and amino acid absorption [37, 38], altered microvascular perfusion and amino acid uptake into muscle [34, 39, 40], and impaired intracellular molecular anabolic signalling [23, 34, 41]. Despite the lack of certainty concerning mechanisms, it is clear that a reduction in the ability of muscle to respond to stimulation from hyperaminoacidaemia is a major factor leading to muscle atrophy with disuse or even reduced activity.

Muscle loss is not the only negative consequence of inactivity in muscle tissue. Muscle mitochondrial oxidative function and metabolic flexibility are impaired with muscle disuse. Downregulation of mitochondrial protein transcription, decreases in translational signalling pathways involved in mitochondrial biogenesis, and declines in mitochondrial enzyme activities all result from immobilization [31]. Some of these changes occur as early as 48 h following initiation of inactivity. Nearly all aspects of mitochondrial function are impacted [31]. It is well-known that muscle disuse leads to depressed insulin sensitivity [42, 43]. Moreover, simply reducing activity of muscle for 2 weeks may lead to decreased insulin sensitivity of muscle [28]. Reduced glucose transport protein 4 (GLUT4) content in immobilized muscle likely contributes to the deleterious impact on glucose metabolism [44]. These adverse changes to muscle oxidative and metabolic function during immobilization are more evidence of the potential for the damaging impact of reduced muscle activity or immobilization following exercise-induced injuries.

## Nutrition Support for Injuries Involving Immobilization and/or Reduced Activity

There are many nutrients and nutritional strategies that have been proposed to help ameliorate the detrimental impact of muscle immobilization and/or decreased activity following injury. The rationale for the use of many nutrients has been touted, yet direct evidence is largely lacking. Only a few studies actually have directly investigated these issues [2, 3]. A complete consideration of all nutrients claimed to confer benefits during muscle loss is beyond the scope of this review. Thus, the discussion will focus on the rationale and evidence for the use of the more prominently evaluated nutrients.

The single most important nutritional consideration during reduced muscle activity and/or immobility is to avoid nutrient deficiencies. Deficiencies of energy, vitamins, minerals and macronutrients—particularly protein—will impair wound healing and exacerbate loss of muscle and tendon mass and function. Whereas healthy exercisers and athletes are unlikely to suffer from malnourishment, choices made during recovery from an injury need to be carefully considered to optimize recovery and return to training.

### Energy

Energy intake is a critical component of any nutrition plan for optimal recovery from an injury resulting in immobilization and reduced activity. However, recommendations for energy intake may not be as obvious as many would believe. Whereas energy intake during short periods of inactivity tends to be greater than expenditure, during prolonged periods of reduced activity, spontaneous energy intake matches energy expenditure [45]. Given that energy expenditure almost certainly will be reduced with a reduction in training and activity, a conscious decision to drastically reduce energy intake is the intuitive choice. If the injured limb is involved in ambulation, energy expenditure may be expected to decline even more, both by necessity and voluntarily due to a reluctance to ambulate [46, 47]. Of course, the amount of alternative training and/or physical activity will be a determinant of total energy expenditure during immobilization. Finally, another, less obvious, contributor to lower energy expenditure is a decrease in protein turnover [21]. Thus, to avoid increased body fat and total mass, most injured athletes, unsurprisingly, will first choose a decrease in energy intake.

The magnitude of any decrease in energy expenditure following an injury with muscle immobilization is likely not as great as was first thought. During the healing process, energy expenditure is increased, particularly if the injury is severe [48]. Energy expenditure may be increased by 15 % up to 50 %, depending on the type and severity of the injury. Thus, whereas reduced physical activity and training may result in reduced total energy expenditure, the overall reduction may be less than appears obvious. Moreover, the energy cost of ambulation may need to be considered. If an athlete must use crutches, the energy expenditure for ambulation is increased two- to threefold [47]. Therefore, the total energy expenditure may not decrease as much as may be at first thought, particularly if the athlete does not voluntarily restrict movement during recovery.

An effort to attain energy balance during recovery from injury is critical. If restriction of energy intake is too severe, recovery almost certainly will be slowed due to negative metabolic consequences. Negative energy balance will interfere with wound healing [48] and exacerbate muscle loss [49, 50]. MPS is an energetically expensive process. It has been estimated that a well-muscled male expends ~500 kcal a day on MPS even without the consideration of physical activity [51]. MPS and associated synthetic intracellular signalling proteins are downregulated by ~20–30 % during even a moderate energy deficit [52, 53]. Given that decreased synthesis of myofibrillar proteins is the major metabolic contributor to muscle loss, if sustained, this energy deficit will result in accelerated loss of muscle mass [49]. Moreover, impaired MPS and negative energy balance, per se, will slow wound healing. Much care should be taken to ensure that sufficient energy is consumed during recovery from an injury.

Whereas, negative energy balance is clearly to be avoided, a large positive energy balance also is undesirable for optimal healing and recovery. Positive energy balance results in increased lean body mass (BM) in healthy humans [54]. Thus, it may be appealing to suggest a positive energy balance during immobilization, even considering a small increase in body fat. However, there is evidence that a positive energy balance actually accelerates muscle loss during inactivity, most likely via activation of systemic inflammation [55]. However, these data stem from a bed rest study, and it is not clear how much systemic inflammation is increased with limb immobility. Moreover, excess energy with reduced activity leads to decreased insulin sensitivity and alterations in muscle and adipose metabolism [56]. Therefore, careful assessment of energy balance via techniques such as indirect calorimetry during both the period of inactivity and rehabilitation may be well worthwhile. It also is possible that energy, per se, may not be the most important factor to consider. The macronutrient composition of the energy may be an operative factor. Recent evidence suggests that oversupply of lipids decreases insulin sensitivity and impairs the response of MPS to amino acids [57]. Thus, both energy and macronutrient intake must be considered very carefully. If reduced energy intake is warranted, factors promoting satiety despite a reduced energy intake, including protein dose and type, plus low energy density choices such as vegetables need to be considered [58]. Energy balance should be the aim during reduced inactivity and/or immobilization due to injury.

### Protein and Amino Acids

The macronutrient most prominently associated with nutrition support for injuries involving immobility is protein. Given a reduction in overall energy intake, if protein intake is kept proportional, an absolute reduction in protein intake is likely. Clearly, insufficient protein intake will impede wound healing and increase inflammation to possibly deleterious levels [59, 60]. Given that muscle loss results from decreased synthesis of myofibrillar proteins [23], and that the healing processes are heavily reliant on synthesis of collagen and other proteins [15], the importance of protein should be obvious. Moreover, the reduction in protein intake, per se, may have a detrimental impact on muscle metabolism—even if the overall intake remains at or near the recommended dietary allowance (0.8 g protein/day/kg BM). This disruption may be particularly evident if habitual protein intake is high, e.g. >1.5 g protein/day/kg BM. A drastic decrease in protein intake results in negative nitrogen balance [61]. During negative energy balance, this loss of nitrogen is almost certainly from muscle [52]. We recently demonstrated that athletes consuming relatively high protein intakes (~2.3 g protein/day/kg BM) had reduced muscle loss during periods of negative energy balance compared with athletes with lower protein intakes (~1.0 g/day/kg BM) [50]. Thus, it may be that relatively high protein intakes, i.e. >2.0 g protein/day/kg BM, are necessary to prevent muscle loss. However, it should be considered that no direct comparison of 1.6 g/day/kg BM to the higher protein intake during energy restriction was made [50]. Consequently, it is not clear if the preservation of muscle in our study was due to increasing the protein intake from habitual (~1.6 g/day/kg BM) to higher intakes in the high protein group or reducing from habitual to the lower amount of protein in the low-protein group. Moreover, during bed rest, increasing protein intake from 1.0 to 1.6 g protein/day/kg BM failed to attenuate muscle loss [62]. A potential contributor to the difference between these studies is that the participants in the bed rest study were female [62]. The influence of sex on the response of muscle to disuse and protein ingestion remains to be elucidated. Nevertheless, it seems clear that appropriate evaluation of habitual protein intake that helps inform recommendations for protein intake after injury should be made.

Other factors in relation to protein should be considered in addition to the absolute amount of protein intake. The pattern of protein intake in terms of timing and amount in each meal is an important factor. The importance of protein intake stems from the resulting hyperaminoacidaemia and increased MPS [63, 64]. In healthy, active muscle ~20–25 g (0.25–0.30 g/kg BM) in one dose of protein maximizes the response of MPS in both resting and exercised muscle [64, 65]. However, given the onset of anabolic resistance with immobility and reduced activity [23, 34, 35], it is likely that the amount of protein in each dose necessary to maximally stimulate MPS in immobilized muscle will be increased [66]. Moreover, the overall response of MPS throughout the day is optimized when this amount of protein is spread equally over the day [67, 68]. This evenly spaced protein intake pattern is markedly different from the pattern habitually used by most athletes [69, 70]. Thus, whereas the impact of meal pattern on the response of MPS during reduced activity is unknown, it seems prudent to recommend that athletes should plan their meal pattern to optimize MPS and ameliorate the loss of muscle protein.

The response of MPS to protein ingestion stems from the EAA content of the protein i.e. nonessential amino acids are not necessary for maximal stimulation of MPS [71, 72]. Thus, EAA supplementation has been recommended for amelioration of muscle loss during muscle disuse following injury. During prolonged bed rest [73] and joint immobilization [74], EAA supplementation has been shown to reduce the loss of muscle mass and strength. However, the dose of EAA may be critical. Smaller doses of EAA failed to prevent muscle loss during bed rest [75]. The volunteers in that study were in negative energy balance. So, it is unclear if the smaller dose of EAA may have been more effective during energy balance. Moreover, unlike many other proposed interventions, there has been direct measurement of muscle loss with EAA supplementation following an injury. Dreyer et al. [76] demonstrated that 20 g of EAA ingested twice daily between meals for 1 week prior and 2 weeks following total knee arthroplasty enhanced recovery in older patients. Of course, it is not certain that injured athletes would experience a similar response to EAA ingestion after injury. Thus, whereas there is not a complete lack of equivocation, there is at least some evidence of efficacy of EAA supplementation during immobilization. Moreover, it is not clear if EAA supplementation is more effective than consuming whole proteins containing the same amount of EAA. Given the cost (and taste) of EAA supplements, intact proteins may be preferred.

The potential for EAA supplementation to ameliorate muscle loss during disuse may be attributed to the branched-chain amino acid leucine, as it has long been known to increase protein synthesis in rodent and cell models [77, 78]. Moreover, recent evidence suggests that leucine ingestion increases MPS in healthy humans [79]. Thus, the use of leucine to ameliorate muscle loss is often touted [80]. However, the impact of leucine on human MPS and muscle loss during disuse is less clear. Leucine has been shown to restore impaired MPS in rats [81, 82] and ameliorates muscle loss in rats during immobilization [83]. Furthermore, supplementation of branched-chain amino acids attenuated the nitrogen loss during bed rest, but did not impact MPS [84]. However, it is possible that leucine may be more effective by overcoming the resistance of muscle to anabolic stimulation. MPS was measured in the fasting state in the bed rest study [84], so there was no assessment of the impact of leucine on anabolic resistance. In older humans, increasing the amount of leucine restored the response of MPS to protein ingestion [85, 86]. Moreover, leucine ingestion increases the utilization of ingested amino acids for MPS [87]. Thus, leucine could play an important role in situations with limited energy and protein intake, such as with injuries. Nevertheless, to date no study has directly investigated the response of muscle to leucine (or branched-chain amino acid) ingestion during a period of muscle disuse following an injury in humans. There are also potential negative effects with use of high-dose leucine supplementation. Thus, caution is warranted prior to making recommendations for leucine supplementation during muscle disuse. Clearly, the evidence is intriguing and this intervention should be attempted in future studies.

### Other Nutrients

There is a theoretical rationale for the efficacy for increased consumption of a variety of nutrients other than protein and amino acids during immobilization or reduced activity following injury. These nutrients include, but are not limited to, creatine, omega-3 fatty acids, and antioxidants. Again it must be emphasized that deficiencies of these nutrients, and others, will impair wound healing and slow recovery. However, evidence that supplementation of nutrients on top of an ample supply will enhance recovery from injury is scarce.

Creatine supplementation is widely used to enhance muscle gains during resistance exercise training [88]. Furthermore, creatine supplementation has been shown to counteract disorders of muscle [89]. However, the evidence for use of creatine to counter muscle loss during immobility is less clear. Creatine supplementation during 2 weeks of lower-limb immobility in otherwise healthy volunteers did not lessen the loss of muscle mass or strength in healthy volunteers during 2 weeks of casting [90]. Moreover, muscle strength was not improved by creatine supplementation following total knee arthroplasty [91]. On the other hand, muscle atrophy in immobilized arm muscle was decreased with creatine supplementation [92]. Thus, it could be that arm and leg muscles respond differently to creatine supplementation during immobility. Moreover, creatine supplementation did prevent a decrease in GLUT4 content during immobilization but increased it to a greater extent than placebo during rehabilitation [44]. Thus, despite questions about the impact on muscle atrophy, creatine may have a positive impact on the muscle oxidative impairments observed during muscle disuse [31, 42–44]. During rehabilitation after immobility, creatine supplementation resulted in an increased rate of muscle growth and strength gains compared with placebo [90]. Thus, the efficacy of creatine supplementation for augmentation of muscle hypertrophy seems to be a consistent finding, but results of investigations on creatine and muscle atrophy are more equivocal.

Omega-3 fatty acids (n-3FA) also have received considerable attention in the context of nutritional support for injuries. In many cases, this attention is related to the anti-inflammatory and immunomodulatory properties of n-3FA [93, 94]. High levels of n-3FA are found in many foods, particularly some cold-water dwelling fish (e.g. mackerel, salmon). Thus, fish oil supplementation is often touted for reduction of inflammation. Supplementation with n-3FA certainly may be important if inflammation is excessive or prolonged [93]. However, as mentioned previously, careful consideration of the use of anti-inflammatory nutrients or drugs is necessary given the importance of the inflammatory response for wound healing [18–20]. There is evidence of impaired wound healing with n-3FA supplementation [95, 96]. Thus, an automatic recommendation of n-3FA supplementation for all injuries does not seem wise.

Another potential property of n-3FA that may have relevance for injuries resulting in immobilization or reduced activity has recently been investigated. Rats fed high amounts of fish oil during hind limb immobilization demonstrated less muscle loss than those rats on high corn oil diets [97]. Moreover, 8 weeks of fish oil supplementation increased the response of MPS to hyperaminoacidaemia and hyperinsulinaemia in both older [98] and younger volunteers [99]. The efficacy for fish oil in this context is thought to be due to changes in the muscle membrane lipid composition in relation to intracellular anabolic signalling [98–100]. This preliminary evidence suggests that fish oil supplementation could play a role in the amelioration of muscle loss with disuse. Then again, high fish oil diets inhibited recovery of muscle mass during recovery from hind limb suspension in rodents [101]. Taken together, it seems that whereas high fish oil (n-3FA) consumption may ameliorate muscle loss during a catabolic situation, it does not seem to be effective to enhance muscle hypertrophy. Moreover, the appropriate dose for injured humans has not been established. Thus, wholesale recommendations for fish oil supplementation during immobilization must be considered premature and caution is warranted.

There is a clear association of many micronutrients, such as zinc, vitamin C, vitamin A (and others), with various aspects of wound healing and recovery from injury, including muscle disuse. For example, vitamin C is associated with hydroxyproline synthesis necessary for collagen formation. For most micronutrients the story is similar, i.e. deficiencies should be avoided, but supplementation above sufficiency does not appear warranted. Sufficient calcium and vitamin D during healing from fractures is important for optimal bone formation. Moreover, there is an association of low vitamin D status with impaired recovery from knee surgery [102]. However, there is no clear evidence for the necessity of supranormal micronutrient intakes during recovery from injury [59].

Oxidative damage is often a concern immediately following an injury. Oxidative damage is thought to be a contributing factor for muscle loss, primarily by increasing MPB [103]. Thus, antioxidant compounds, including n-3FA, have been commonly recommended to improve healing and recovery [60, 103]. Antioxidant supplementation in rodent models results in decreased oxidative stress, but equivocal results in terms of muscle loss with immobility [103]. In high doses, there does seem to be some impact of antioxidant supplementation on muscle loss in rodents. However, equivalent doses likely would be problematic and potentially toxic if taken by humans [103]. Lower doses that might be better tolerated tend not to be as effective. In one human study, vitamin C and E supplementation failed to influence recovery of muscle dysfunction following knee surgery [104]. However, vitamin C status prior to supplementation was correlated with improvements in muscle function. Thus, taken together, these results suggest that sufficient antioxidant intake is important for optimal recovery, but supplementation on top of sufficiency is unnecessary if nutrient status is adequate.

## Nutrition Support for Other Injuries

Not all injuries require limb immobilization. So, even if training is curtailed or reduced, muscle loss may be less and the metabolic consequences might not be as severe. There is also evidence that some injuries might have particular nutritional requirements. Thus, a brief discussion of what little is known about nutrition to support a few selected types of injuries seems warranted.

### Concussion/Traumatic Brain Injuries

Traumatic brain injuries (TBI) in athletes are attracting an increasing amount of attention and scrutiny. In contact sports, such as rugby and American football, these injuries are increasingly common. TBI also are common in other contact sports and in military personnel. Diagnosis of TBI is being treated much more seriously than in earlier times. Moreover, increasing awareness of long-term consequences of TBI, particularly if there are repetitive incidences, is forthcoming. Significant brain abnormalities were reported in a group of retired American football players [105]. In addition, retired American football players over the age of 50 with a history of repetitive TBI demonstrated rates of cognitive impairment five times that of retirees without a history of TBI [106]. The pathogenic process leading to these problems is related to the secondary phase of recovery following TBI, which includes processes such as neuroinflammation, increased excitatory amino acids, free radicals and ion imbalances that lead to axonal and neuronal damage [107]. However, there still are no approved therapies to treat TBI or the underlying processes of TBI and enhance recovery from TBI [107]. Thus, it seems clear that a nutritional intervention that could ameliorate the consequences of TBI and improve cognitive and neuromuscular function would be valuable for active and retired athletes.

Nutritional treatments for TBI-related problems centre around antioxidants and anti-inflammatory agents. Virtually all of the research to date is based on rodent models. One study showed that rats eating a diet supplemented with curcumin, an anti-inflammatory compound, had decreased levels of factors found to increase following TBI. These factors include oxidized proteins, normalized brain-derived neurotrophic factor (BDNF), and molecules in the pathogenic pathway downstream of BDNF [108]. Moreover, cognitive function was improved in the rats consuming supplemental curcumin. The efficacy of n-3FA for amelioration of TBI-related damage also has been investigated. Animal studies consistently demonstrated that both prophylactic and therapeutic use of n-3FA decreases axonal and neuronal damage, inflammation, and apoptosis and normalizes BDNF and neurotransmitter levels [109–113]. Moreover, these changes lead to improved cognitive function. Thus, there seems to be promising evidence of the efficacy of curcumin and, especially, n-3FA for recovery from TBI in rodents. However, it is not clear if the efficacy of n-3FA for TBI in rodents can be generalized to humans.

The promising nature of data generated from animal studies suggests that n-3FA may be an effective nutrient to counter the negative long-term effects of TBI. To date, no study has been published examining this question in humans. However, clinical trials are under way after the US Institute of Medicine recommended further investigation in 2011. There have been a small number of case studies suggesting that high-dose n-3FA may improve acute outcomes after TBI [114, 115]. Moreover, an open-design study demonstrated that a nutritional intervention, including n-3FA, improved cognitive function in retired American football players with a history of TBI [116]. However, the players in this study participated in lifestyle interventions in addition to consuming a supplement that contained n-3FA and several other ingredients. Thus, the contribution of the n-3FA, other nutrients, or the lifestyle intervention to the improvement in cognitive function cannot be definitively identified. A follow-up, double-blind, placebo-controlled study determined that nutritional supplementation, including n-3FA, resulted in improved neuropsychological function in healthy volunteers [117]. Again, determination of the precise role of n-3FA in this improvement is not possible given the large number of nutrients consumed in the supplement. Therefore, whereas preclinical and preliminary data on the impact of n-3FA for recovery from TBI are promising, solid recommendations to include n-3FA in a treatment regimen cannot be made, at least until the results of the ongoing clinical trials are reported.

### Muscle Tissue Injuries

Common exercise-induced injuries include those with damaged muscle and other soft tissues. These injuries likely will not necessarily result in immobilization of the limb, but will require a reduction in activity of the injured limb—if for no other reason than that the injury is painful. A common model used to examine muscle injuries is an eccentric exercise model. In this model, the volunteers perform a number of eccentric—force production during muscle lengthening—contractions. Loss of muscle function, increases in blood proteins associated with muscle damage, and increased pain result from these types of situations [118–121]. Several methods have been used to perform the eccentric contractions, including eccentric resistance exercise, dynamometers, stepping down from a bench or block and downhill running [119]. Many investigations have focused on nutrients that may be useful in recovery from these intense exercise situations.

Many nutrients have been touted to alleviate symptoms associated with muscular injuries using these eccentric exercise models. A complete examination of this literature is beyond the scope of this review. Interested readers are referred to recent reviews [118, 121–124]. Overall, the nutrients most often associated with alleviation of pain and increased recovery from eccentric exercise include protein and amino acids, anti-inflammatory compounds and antioxidants. The available information does not readily lend itself to a solid conclusion for any of these nutritional countermeasures to the deleterious effects of muscle injury. Moreover, it is not certain that this eccentric exercise model is an appropriate way to evaluate soft tissue injuries in exercisers. Nonetheless, many studies have attempted to assess nutritional interventions to enhance recovery after exercise-induced muscle damage and many recommendations are commonly made.

Protein and amino acids probably have been the most widely studied nutrients in the context of muscle injuries. There are studies suggesting that protein [125] and/or free amino acids [126] may alleviate some indicators of muscle damage. Any positive impact on recovery may be due to the branched-chain amino acid content of the protein [127, 128]. The impact of protein has been attributed to increased MPS to enhance repair [124]. However, changes in indices of muscle damage occur in the order of hours [124, 127, 128]. Given that the turnover of structural muscle proteins is quite slow [26, 129], it is difficult to accept this attribution. Moreover, other studies do not report an effect of protein or amino acids [130, 131]. The variable results are likely due to varying supplementation patterns, types of exercise, and other design considerations [124]. Finally, many of the volunteers in the studies were untrained and generalizability of the results to an athletic population may be questioned. Thus, this equivocality makes it difficult to conclude that protein or amino acid supplementation enhances recovery from muscle injury, particularly for injured athletes. In fact, a recent systematic review concluded that the evidence for alleviation of symptoms of muscle injury by protein and amino acids is lacking [124].

Provision of antioxidants and anti-inflammatory agents to alleviate symptoms of muscle damage also has been a popular strategy. However, at best, as with protein, the literature can only be considered equivocal. The interested reader is referred to recent reviews [121, 123] for a more detailed examination of these studies. Clearly, given the disparity in the types of exercise, supplementation patterns, and other methodological issues, very little insight into nutrition for muscle injuries can be gleaned from exercise-induced muscle damage studies. Hence, it is not possible to make solid recommendations regarding nutritional countermeasures to exercise-induced muscle damage and injuries.

## What to Avoid

Thus far, the focus of the discussion has been on what nutrients to consume. However, consideration of what to avoid also should be made. As mentioned above, the most obvious nutrition consideration is to avoid nutrient deficiencies. This consideration was discussed earlier in the context of inactivity and immobilization and should be the number one overriding priority for nutrition related to injury. Additionally, nutrient excess should be avoided. Excess energy could lead to increased total and fat mass, particularly if activity is dramatically reduced. In light of the preliminary evidence for the efficacy of n-3FA in the context of several different injuries that has been discussed, careful consideration of the dose should be made before advising an injured athlete. Excess n-3FA consumption could excessively depress the inflammatory response leading to impaired wound healing [95, 96]. However, studies determining the appropriate or excessive doses in humans have not been conducted. Thus, caution is justified.

One obvious nutrient that is best avoided or at least ingested in only small amounts is alcohol. Alcohol ingestion impairs MPS in rats [132], as well as the response of MPS to exercise in humans [133]. Moreover, it is clear that alcohol impairs wound healing, likely by reducing the inflammatory response [134], and increases muscle loss during immobilization in rats [135]. Thus, whereas it may be self-evident, it is worth emphasizing that limited alcohol ingestion during recovery is important. So, as tempting as it may be to indulge in alcohol to drown sorrows or diminish pain, only small amounts, if any, should be imbibed.

## Summary and Conclusions

In summary, there is much still to be learned about the best nutritional strategy to enhance recovery from exercise-induced injuries. There are claims for the efficacy of many nutrients, yet direct evidence is sorely lacking. It is quite clear that a careful evaluation of each patient’s situation must be conducted. Nutritional status and energy requirements should be assessed throughout recovery and nutrient intake adjusted accordingly. Deficiencies, particularly those of energy, protein, and micronutrients, must be avoided. Energy balance is critical. Higher protein intakes (~2–2.5 g protein/kg BM/day) may be warranted, but at the very least the absolute amount of protein intake should be maintained even in the face of reduced energy intake. There is promising—albeit it must be considered preliminary—evidence for the efficacy of other nutrients in certain situations. Leucine, n-3FA, curcumin, and others have been demonstrated to be beneficial in rodent studies, but information from studies on injured humans is yet forthcoming. In some situations, higher intakes of these nutrients may do harm. Moreover, even if they are efficacious for injured humans, there is no information regarding the optimal dose of these nutrients. Thus, caution is warranted before recommendations for wholesale use of these nutrients by injured athletes are made.

The best recommendation would be to adopt a ‘first, do no harm’ approach. The use and amount of each nutrient should be considered in the context of a risk/benefit ratio. Even if the benefit is uncertain, it may be worth trying if no risks can be identified. Otherwise, if there is a risk of doing harm with use of a particular nutrient, then perhaps that nutrient should be avoided. As always, the basis of nutritional strategy for an injured exerciser should be a well-balanced diet based on a diet of whole foods from nature (or foods made from ingredients from those foods) that are minimally processed [136]. Whereas this advice may be considered mundane, boring, and lacking insight, it seems still to be the best course of action.
